# Stent thrombosis associated with non-response to clopidogrel despite co-administration of aspirin and ibrutinib

**DOI:** 10.1093/ehjcr/ytag352

**Published:** 2026-05-21

**Authors:** Omar Dirir, William A E Parker, Hazel A Haley, Julian P Gunn, Robert F Storey

**Affiliations:** Cardiovascular Research Unit, Division of Clinical Medicine, The University of Sheffield, Sheffield S10 2RX, UK; NIHR Sheffield Biomedical Research Centre, Sheffield Teaching Hospitals NHS Foundation Trust, Sheffield S10 2JF, UK; Cardiovascular Research Unit, Division of Clinical Medicine, The University of Sheffield, Sheffield S10 2RX, UK; NIHR Sheffield Biomedical Research Centre, Sheffield Teaching Hospitals NHS Foundation Trust, Sheffield S10 2JF, UK; South Yorkshire Cardiothoracic Centre, Sheffield Teaching Hospitals NHS Foundation Trust, Sheffield S5 7AU, UK; South Yorkshire Cardiothoracic Centre, Sheffield Teaching Hospitals NHS Foundation Trust, Sheffield S5 7AU, UK; Cardiovascular Research Unit, Division of Clinical Medicine, The University of Sheffield, Sheffield S10 2RX, UK; NIHR Sheffield Biomedical Research Centre, Sheffield Teaching Hospitals NHS Foundation Trust, Sheffield S10 2JF, UK; South Yorkshire Cardiothoracic Centre, Sheffield Teaching Hospitals NHS Foundation Trust, Sheffield S5 7AU, UK; Cardiovascular Research Unit, Division of Clinical Medicine, The University of Sheffield, Sheffield S10 2RX, UK; NIHR Sheffield Biomedical Research Centre, Sheffield Teaching Hospitals NHS Foundation Trust, Sheffield S10 2JF, UK; South Yorkshire Cardiothoracic Centre, Sheffield Teaching Hospitals NHS Foundation Trust, Sheffield S5 7AU, UK

**Keywords:** Stent thrombosis, Clopidogrel non-responder, Platelet function testing, Tyrosine kinase inhibitor, Ibrutinib, Case report

## Abstract

**Background:**

Managing atherosclerotic cardiovascular disease in individuals with chronic lymphocytic leukaemia (CLL) is complicated by the uncertain antiplatelet effect exerted by Bruton’s tyrosine kinase inhibitors. Concerns over increased bleeding risk with concurrent ibrutinib and dual antiplatelet therapy (DAPT) are balanced with the need to provide robust antiplatelet cover following percutaneous coronary intervention (PCI) and reduce the risk of stent-related complications.

**Case summary:**

We describe a case of an 83-year-old patient with a background of stable CLL on ibrutinib. He was appropriately established on DAPT (clopidogrel and aspirin) prior to elective PCI. Post-procedure, the patient re-presented after six days with subacute stent thrombosis (SST). Platelet function tests demonstrated clopidogrel non-responder status, with high platelet reactivity to multiple agonists. He was switched to ticagrelor with excellent antiplatelet response and underwent repeat PCI.

**Discussion:**

This case is the first description of SST in a patient on concurrent DAPT and ibrutinib. It highlights the need for appropriately robust P2Y_12_ inhibition despite bleeding concerns and ex vivo studies demonstrating some antiplatelet effect by ibrutinib. This case also highlights the role for platelet function testing to tailor antithrombotic regimens in complex and high-risk PCI patients.

Learning points

- Stent thrombosis can be a life-threatening complication of PCI and is often related to insufficient antiplatelet therapy as well as suboptimal stent implantation
- Ibrutinib provides limited platelet inhibition that is insufficient in the post-PCI period
- Platelet function testing allows for rapid assessment of antithrombotic regimens and intravascular imaging may aid stent optimization in complex and high-risk PCI patients

## Introduction

Ibrutinib is an oral irreversible Bruton’s tyrosine kinase (Btk) inhibitor licensed for long-term use in chronic lymphocytic leukaemia (CLL). Btk is involved in downstream signalling transduction following the formation of collagen-glycoprotein (GP)VI complexes, a principal step in platelet-mediated arterial thrombosis.^1^ This has led to optimism in ibrutinib having a potential role as an antiplatelet agent.^2,3^ However, ibrutinib is associated with increased bleeding risk^4^ and its impact in the context of cardiovascular disease and concurrent dual antiplatelet therapy (DAPT) is poorly understood.^5^ We describe a case of subacute stent thrombosis (SST) post-percutaneous coronary intervention (PCI) in a patient with CLL despite treatment with ibrutinib, aspirin and clopidogrel.

## Summary figure

**Figure:**
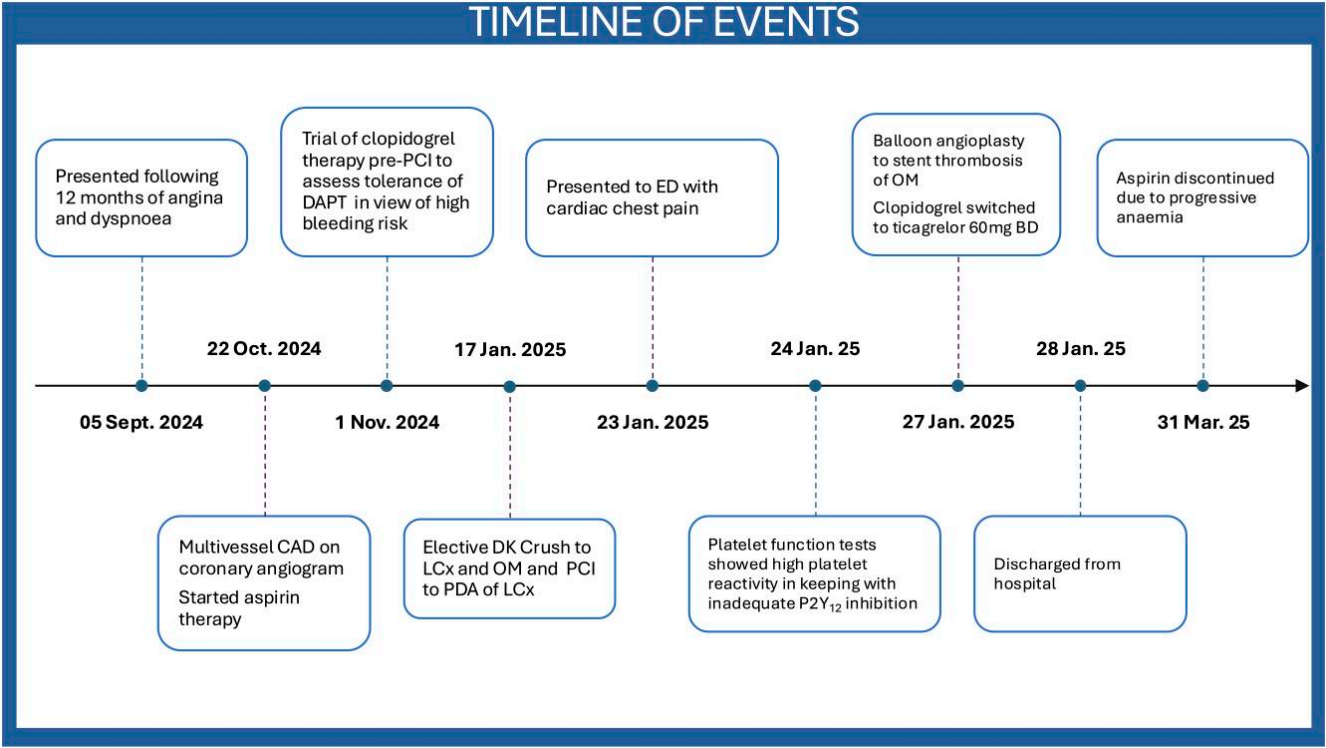


## Case presentation

**Figure 1:**
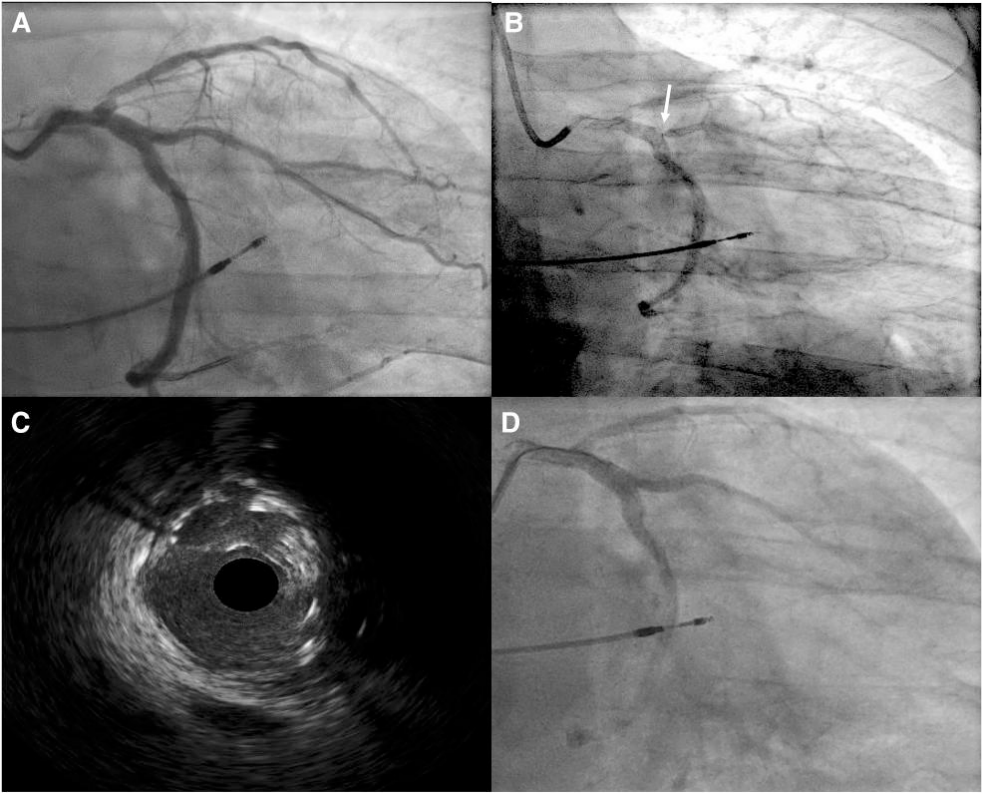
Coronary angiogram post-index PCI (*A*), the SST identified via coronary angiography (*B*) and intravascular ultrasound (*C*) and following angioplasty of the SST (D).

An 83-year-old gentleman with a background of CLL on long-term ibrutinib 420 mg once daily, moderate-to-severe aortic stenosis (valve area 1.22 cm^2^) and stable angina underwent diagnostic coronary angiography in October 2024 that identified moderate distal left main stem disease and three-vessel coronary artery disease (see Supplementary Materials, Supplementary material online, *Video S1*). His past medical history included permanent pacemaker for 2:1 atrioventricular block, ischaemic stroke in 2010, hypertension, stage 3a chronic kidney disease, and laparoscopic hemicolectomy for colorectal cancer (pT3N1bM0) in 2014. He was commenced on aspirin 75 mg once daily in October 2024 and subsequently on clopidogrel 75 mg once daily in November 2024 to assess tolerance of DAPT in view of his high risk of bleeding. Two months after commencing clopidogrel, he underwent PCI to his left circumflex (Cx) artery and obtuse marginal (OM) bifurcation using the ‘DK crush’ technique, receiving stents to the proximal Cx and OM, as well as PCI with further stent to the posterior descending artery of the Cx (*Figure 1A*, Supplementary material online, *Video S1*). There were no immediate complications and he was discharged the same day. On the advice of the Haematology team, his ibrutinib was withheld five days prior to and restarted 24 h after his procedure.

**Figure 2:**
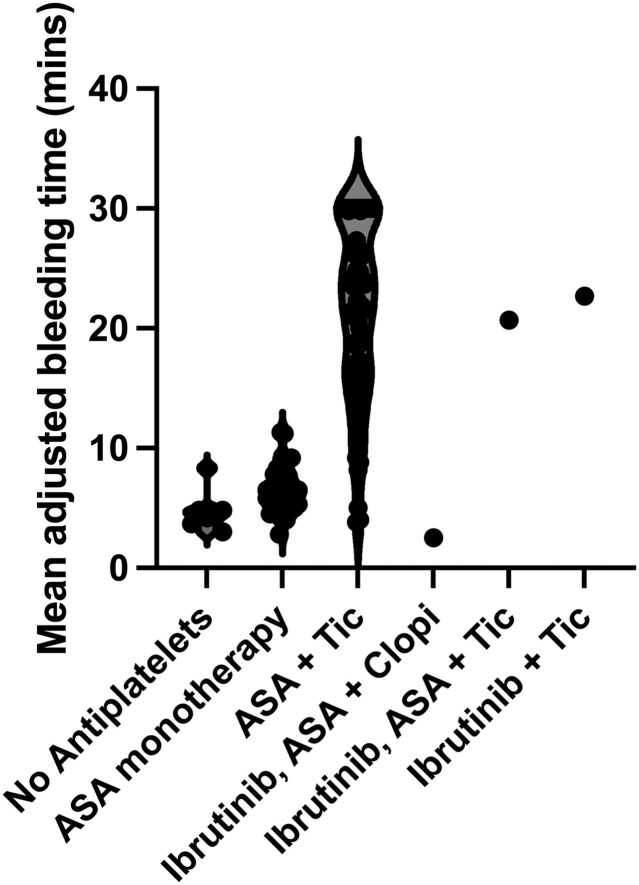
Violin plot of mean adjusted bleeding time for individuals; not on antiplatelet therapy; on aspirin (ASA) monotherapy; dual antiplatelet therapy (DAPT) consisting of ticagrelor (Tic) and aspirin; and the patient whilst on combinations of aspirin, clopidogrel (Clopi) and ticagrelor 60 mg bd.

**Figure 3:**
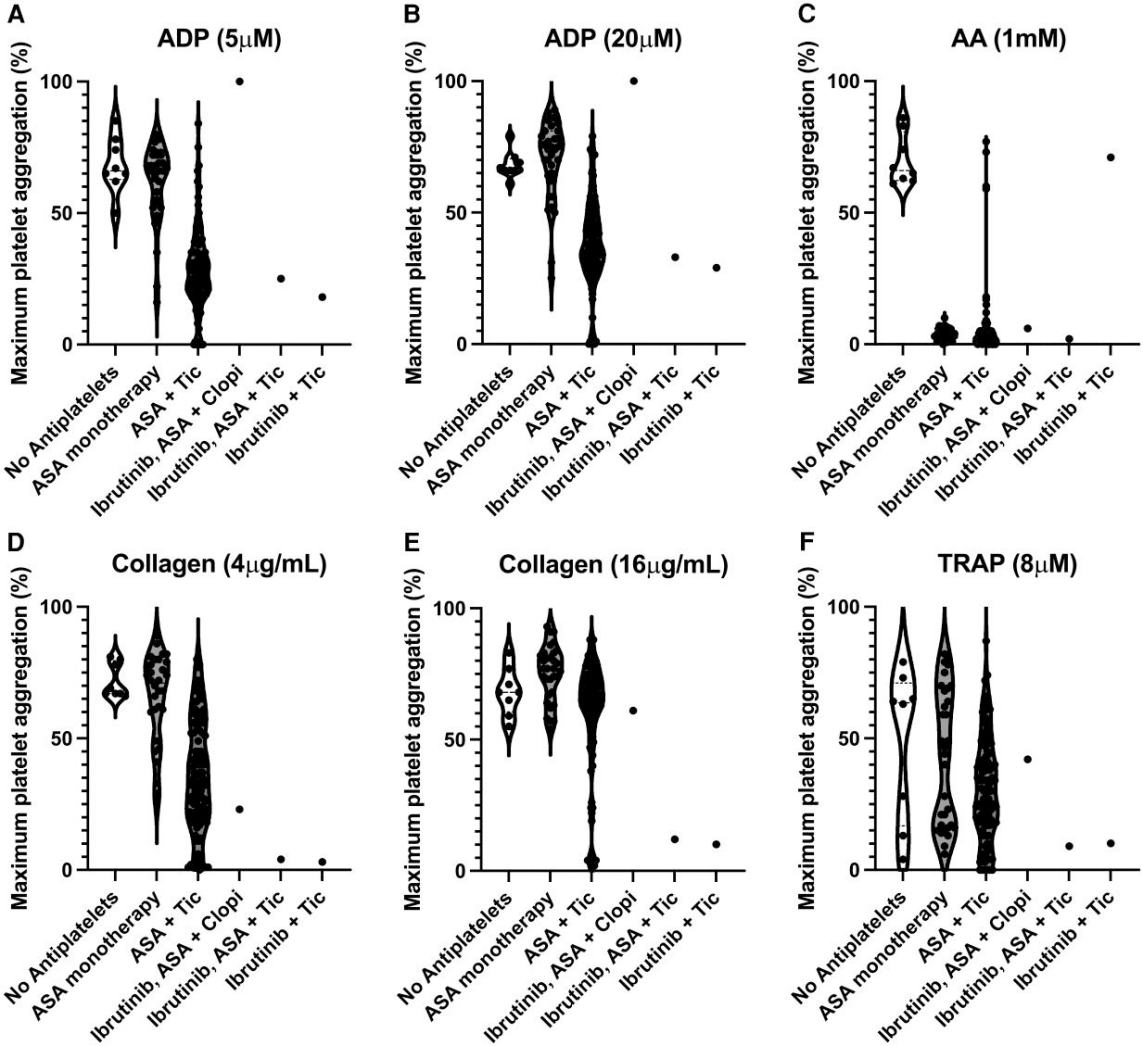
Maximum platelet aggregation (%) assessed by light transmission aggregometry in response to (*A*) low-concentration and (*B*) high-concentration adenosine diphosphate (ADP), (*C*) arachidonic acid (AA), (*D*) low-concentration, and (*E*) high-concentration collagen, (*F*) thrombin-related activating peptide (TRAP) in individuals; not on antiplatelet therapy; on aspirin (ASA) monotherapy; dual antiplatelet therapy (DAPT) consisting of ticagrelor (tic) and aspirin; and the patient whilst on combinations of aspirin, clopidogrel (clopi) and ticagrelor 60 mg bd.

He presented to the hospital six days later with cardiac chest pain. Clinical examination revealed stable vital signs and minimal lower limb oedema, with a subsequent plain film chest radiograph showing clear lung fields. His ECG showed no new changes. Serum high-sensitivity troponin-T level was 205 ng/L, rising to 219 ng/L after six hours. During this admission, he was enrolled in an observational study of markers of thrombotic risk in patients with, or at increased risk of, cardiovascular disease, according to a protocol approved by the National Research Ethics Service and Health Research Authority that included not conveying the test results to the clinical care team for clinical decision-making. A bedside bleeding time test showed an unexpectedly short time to haemostasis (2.5 min) despite compliance with DAPT and ibrutinib (*Figure 2*). Subsequent platelet function tests demonstrated complete absence of response to clopidogrel with an exaggerated platelet aggregation response (100%) on light transmission aggregometry to both a low concentration (5 μM) and a high concentration (20 μM) of adenosine diphosphate (ADP) (*Figure 3A* and *B*). Similarly, high platelet reactivity was observed in response to high-concentration thrombin-receptor-activating peptide (TRAP) (*Figure 3F*). Conversely, platelet responses to low-concentration (4 μg/mL) and high-concentration (16 μg/mL) collagen were consistent with expected results for those on DAPT (*Figure 3D* and *E*), suggesting a modest effect of ibrutinib on collagen-induced platelet aggregation, considering the lack of inhibition of ADP-induced platelet aggregation.

Coronary angiography confirmed the diagnosis of SST (*Figure 1B*, Supplementary material online, *Video S2*), which was treated successfully with balloon angioplasty guided by intravascular ultrasound (IVUS) (*Figure 1C* and *D*, Supplementary material online, *Video S3* and Supplementary Materials  *Video S2*). Clopidogrel was discontinued and the patient was loaded on ticagrelor 180 mg followed by a maintenance dose of 60 mg twice daily. Repeat bleeding time increased to 20.7 mins and platelet function tests demonstrated a high level of platelet P2Y_12_ inhibition (*Figures 2* and *3*). He had no further thrombotic events but required cessation of aspirin after 2 months due to progressive anaemia. His haemoglobin subsequently stabilized at 111 g/L, whilst bedside bleeding time remained consistent with appropriate antiplatelet therapy (22.7 min).

Data-sharing statement: The data underlying this article are available in the article.

## Discussion

Atherosclerotic plaque injury exposes circulating platelets to thrombogenic collagen fibres, resulting in GPVI receptors binding to collagen^1^ and platelet activation and aggregation, via Btk signalling.^5^ Btk’s role in this pathway has previously identified ibrutinib as a potential antiplatelet agent.^2^ However, this case of a clopidogrel non-responder has demonstrated that ibrutinib may provide insufficient platelet inhibition post-PCI.

It is likely that high-platelet reactivity (HPR) was a major causative factor in the outcome of SST, combined with lack of IVUS-guided optimization of stent deployment in the index PCI procedure. HPR in clopidogrel-treated patients is commonly but not exclusively associated with loss-of-function mutations in *CYP2C19*, the gene encoding the cytochrome P450 enzyme that converts clopidogrel to its active metabolite.^6^ We did not determine *CYP2C19* genotype in this case but have previously noted that absence of *CYP2C19* loss-of-function alleles does not necessarily avoid poor pharmacodynamic response to clopidogrel.^7^ Approximately one-third of patients are clopidogrel low-responders and the consequent HPR is associated with recurrent ischaemic events and stent-related complications.^8^ Uninhibited ADP-mediated P2Y_12_ receptor activation results in sustained glycoprotein IIb/IIIa receptor activation, release of prothrombotic platelet granule contents and platelet procoagulant activity.^9^ Ticagrelor consistently provides a high degree of platelet inhibition, including in clopidogrel non-responders.^7,10^ Platelet function testing could be key in identifying such individuals to improve clinical outcomes by facilitating personalized antithrombotic regimens, such as escalation of antiplatelet therapy in clopidogrel-treated patients who have high risk of stent thrombosis.^11^

The absence of intravascular imaging (IVUS or optical coherence tomography) during the index PCI potentially increased the risk of stent-related complications. Bifurcation lesions requiring complex PCI, such as in this case, are associated with increased rates of complications.^12^ The use of intravascular imaging supports good stent expansion and apposition, reducing the risk of post-procedure complications.^13^

Platelet aggregation following collagen stimulation was similar to that seen in the context of DAPT (*Figure 3D* and *E*), implying some inhibition by ibrutinib in the context of absent P2Y_12_-inhibitory effect of clopidogrel. This is in keeping with ibrutinib’s mechanism of action^14^ and suggests appropriate therapeutic levels of ibrutinib although these were not measured. Maximal platelet aggregation following stimulation with ADP and TRAP demonstrated both insufficient P2Y_12_ inhibition and that ibrutinib does not appear to inhibit non-collagen-mediated platelet activation pathways, raising concerns about the notion of ibrutinib being utilized as an alternative to either aspirin or a P2Y_12_ inhibitor for prevention of SST.

## Conclusion

This case highlights the role that platelet function testing might play in providing personalized antithrombotic regimens in patients with complex co-morbidities and bleeding concerns. In this case, conventional clinical reasoning would favour the use of clopidogrel in view of a previous stroke and concerns over an increased bleeding risk.^15^ However, platelet function testing indicated complete absence of response to clopidogrel and insufficient compensatory antiplatelet effects of ibrutinib, which were likely significant factors in the development of SST. More widespread platelet function testing in clinical practice could improve outcomes through personalized antiplatelet therapy and this warrants further assessment.

## Supplementary Material

ytag352_Supplementary_Data

## Lead author biography

Dr Omar Dirir is a clinical research fellow in the Cardiovascular Research Unit at the University of Sheffield. He undertakes his clinical work in the Cardiology Department of Sheffield Teaching Hospitals NHS Foundation Trust. He graduated from Sheffield Medical School with bachelor’s degree in medicine and surgery and a first-class honours degree in medical sciences. He is currently undertaking a doctorate in cardiovascular science, focussing on thrombosis and haemostasis in atherosclerotic cardiovascular disease.

## Notes

**Consent:** The patient described in this case report provided written informed consent for the publication of the details contained in this report, consistent with Committee on Publication Ethics guidelines.
